# Basal ganglia calcification as a putative cause for cognitive decline

**DOI:** 10.1590/S1980-57642013DN70200003

**Published:** 2013

**Authors:** João Ricardo Mendes de Oliveira, Matheus Fernandes de Oliveira

**Affiliations:** 1 Neuropsychiatric Department - Federal University of Pernambuco (UFPE), Recife PE, Brazil.; 2 Neurosurgery Residency Program. Hospital do Servidor Público Estadual de São Paulo, São Paulo SP, Brazil.

**Keywords:** basal ganglia, dementia, calcinosis

## Abstract

Basal ganglia calcifications (BGC) may be present in various medical conditions, such as infections, metabolic, psychiatric and neurological diseases, associated with different etiologies and clinical outcomes, including parkinsonism, psychosis, mood swings and dementia. A literature review was performed highlighting the main neuropsychological findings of BGC, with particular attention to clinical reports of cognitive decline. Neuroimaging studies combined with neuropsychological analysis show that some patients have shown progressive disturbances of selective attention, declarative memory and verbal perseveration. Therefore, the calcification process might represent a putative cause for dementia syndromes, suggesting a probable link among calcinosis, the aging process and eventually with neuronal death. The increasing number of reports available will foster a necessary discussion about cerebral calcinosis and its role in determining symptomatology in dementia patients

## Abstract (pt)

As calcificações dos gânglios da base (BGC) podem estar presentes em diversas condições médicas, como infecções, doenças metabólicas, psiquiátricas e neurológicas, associadas a diferentes etiologias e desfechos clínicos, incluindo parkinsonismo, psicose, alterações de humor e demência. Realizamos revisão da literatura destacando os principais achados neuropsicológicos das BGC, com especial atenção para os relatos de declínio cognitivo. Estudos de neuroimagem combinados com análise neuropsicológica mostram que alguns pacientes apresentam distúrbios progressivos de atenção seletiva, memória declarativa e perseverança verbal. Portanto, o processo de calcificação pode representar uma causa imputável para síndromes demenciais, sugerindo uma provável ligação entre a calcinose, o processo de envelhecimento e a morte neuronal. O número crescente de relatos disponíveis promoverá uma discussão necessária sobre calcinose cerebral e seu papel na determinação da sintomatologia em pacientes demenciais.

## INTRODUCTION

asal ganglia calcifications (BGC) may be present in various medical conditions, such as infections, metabolic, psychiatric and neurological diseases, associated with different etiologies and clinical outcomes, including parkinsonism, psychosis, mood swings and dementia.^1,2^ BGC may be present in up to 12% of patients screened with a routine Skull tomography (CT).^3^ Nosologic distinction is made between the idiopathic form (also called Fahr's disease), when there is no clear cause for the symptoms; and the secondary form when a known disorder such as endocrinopathy or infection is identified.^2^

The aim of this paper was to describe the most important clinical aspects of BGC and focus on cognitive impairment symptoms, especially in its idiopathic form

## METHODS

A literature review was carried out involving the main scientific Databases, including Pubmed, Lilacs and Scielo, searching for the terms "basal ganglia calcifications", "idiopathic basal ganglia calcifications", "cognitive" and "neuropsychology". All clinical reports were considered according to the description of basal ganglia calcifications and any feature of neuropsychological impairment

## RESULTS

**Generalisation.** BCG is usually diagnosed in individuals between 30 and 60 years old. Some authors reported different clinical forms of BGC according to the age of onset for symptoms: a childhood form, with precocious manifestation in the first years of life, an early adult onset type with presentation between 20 and 40 years of age, manifesting as a schizophrenia-like psychosis and finally a late-onset variety, presenting between the ages of 40 and 60, associated with dementia and parkinsonism.^4,5^ However, the study of familial BGC shows that clinical heterogeneity is very common even among relatives, developing symptoms, sometimes, in those three different moments of life.^6-9^

Neuropathological analysis suggests that basal ganglia calcinosis implicate in neuronal loss, hypometabolism, hypoperfusionand impairment of neurochemmical activity. A connectivity impairment of basal ganglia with frontal lobe, corticobasal structures and interhemispheric relations, generates psychiatric, cognitive, and behavioral changes.^1,10-12^ Depletions in variable grades in dopamine, glutamate and acetylcholine synapses are the main chemical findings. Limbic structures and temporal lobe may also be enrolled, leading to mood swings and memory disturbances.^1,10-12^

**Neuropsychological and cognitive assessment.** Neuropsychological background tests in a 50-year-old medical doctor evidenced severe deficits in mental flexibility, problem solving and planning, as well as in verbal memory, suggesting participation of the dorsolateral prefrontal loop which mediates problem solving and mental flexibility. He showed no deficits in motivation or drive and had socially appropriate behavior during testing. He also showed intact language abilities and verbal intelligence in the upper average range.^13^

Palilalia and dysarthria might be found as a language impairment.^11^ Idiopathic BGC (IBCG) has been associated with personality changes due to frontal-subcortical dysfunction.^2^ Lopez-Villegas et al. (1996) described relevant impairments in executive functions, visuospatial skills, and selected memory functions.^14^

Konupcíková et al. (2008) reported a case of a 62year-old man presenting extensive brain calcifications, including BGC but with mild movement disorders , mild calcium metabolism abnormalities and severe dementia.^15^ Weisman et al. (2007) also described the case of a 66-year-old man, without any metabolic alterations but with diffuse brain calcification and progressive dementia for 5 years, showing early signs of frontal impairment.^16^

Some patients present a combined motor and cognitive impairment, such as the patient reported by Paschali et al. (2009), a 56-year-old woman with IBGC ,and showing progressive gait disturbances, reduced psycho-motor speed, speech difficulty and memory impairment. Reduced glucose uptake in PET in basal ganglia, frontal, temporal and parietal cortex was also detected. The Dopamine transporter (DAT) SPECT/TC combined with a low-dose x-ray computerized tomography transmission (hybrid SPECT/CT) and 99^m^Tc-D,L hexamethylpropylene amine oxime (99^m^Tc-HMPAO) revealed a reduction in DAT binding in both striation regions coinciding with bilateral calcifications in the basal ganglia, correlating well with the clinical condition of the patient.

Comorbidity with unusual conditions should also be considered and there is a particular report of a case of a patient with previous poliomyelitis coexisting with IBGC.^18^ Severe and diffuse cognitive impairment at age 67 was consistent with the diagnosis of dementia in the patient. He had severe executive dysfunction with an attention deficit, intrusions, perseverations, reduced verbal fluency and loss of cognitive flexibility. Working memory was impaired, as well as visual and verbal episodic memory, judgment and performance on visuospatial tasks.^18^

**Neuroimaging studies.** Computerized tomography is the best exam to detect brain calcifications but neuroimaging studies combined with neuropsychological analysis showed that some patients present progressive disturbances of selective attention, declarative memory and verbal perseveration.^19^

A reduced uptake in striatum bilaterally, the frontal, temporal and parietal cortices was detected through PET in a patient presenting with BGC, hyperkinetic-hypotonic syndrome, impaired cognitive flexibility, figural memory and constructive praxis.^19^

By contrast, reduced glucose uptake in PET was not only restricted to the left basal ganglia, but also involved the right temporoparietal and the right cerebellar cortices corresponding to the impaired cognitive flexibility, figural, and working memory.^20^

In another familial study, eight patients were analyzed over 3 generations with IBGC detected on brain CT scan, showing an autosomal dominant mode of inheritance and predominant psychiatric and cognitive impairment, with 3 of these members also undergoing a FDG brain metabolic analysis. Cortical dysfunction might be detected early in patients with idiopathic BGC, suggesting that retrosplenial hypometabolism might be involved in the episodic memory deficit.^21^

A FDG PET brain scan in a 50 year-old man presenting with a 6-month history of personality change and impairment of planning and memory functions showed that significant reductions of glucose metabolism in basal ganglia and frontal lobes, particularly the dorsolateral, orbitofrontal, and ventromedial cortex. No significant hypometabolism was seen in the temporal or parietal cortex.^22^

## DISCUSSION

Basal ganglia calcification (BGC) in its idiopathic or secondary form is more classically labeled as a subcortical cause of dementia. Cognitive and behavioral findings might also include mood disorders, psychosis, mood swings, with significant impairment on tests of frontal-executive functions, resembling other degenerative diseases with basal ganglia dysfunction.^1,2^ A marked presence of motor symptoms, especially a rigid hypo-kinetic syndrome, helps to differentiate this condition from other subcortical syndromes.^10^

Therefore, the calcification process might represent a putative cause for dementia syndromes, suggesting a probable link between calcinosis, the aging process and eventually with neuronal death.^6-9^

The increasing number of reports available will foster a necessary discussion about cerebral calcinosis and its role in determining symptomatology in dementia patients.

## Figures and Tables

**Table 1:** Main neuropsychological findings in basal ganglia calcifications patients.

Author	Neuropsychological and cognitive profile
Delazer^13^	Deficits in mental flexibility, problem solving and planning, verbal memory
Lopez-Villegas^14^	Impairments in executive functions, visuospatial skills, and selected memory functions
Konupcíková^15^	Severe demential state
Weisman^16^	Progressive dementia
Paschali^17^	progressive gait disturbances, memory impairment, speech difficulty, and reduced psychomotor speed
Hempel^19^	disturbances of selective attention, verbal perseveration and declarative memory
Geschwind^2^	impaired cognitive flexibility, figural, and working memory
Cartier^10^	Impaired verbal and visual-spatial memory, planning, attention and concentration capacities and visual constructive skills
